# Method development in quantitative NMR towards metrologically traceable organic certified reference materials used as ^31^P qNMR standards

**DOI:** 10.1007/s00216-014-8306-6

**Published:** 2014-11-22

**Authors:** Michael Weber, Christine Hellriegel, Alexander Rueck, Juerg Wuethrich, Peter Jenks, Markus Obkircher

**Affiliations:** Sigma-Aldrich Switzerland, Industriestrasse 25, 9471 Buchs, Switzerland

**Keywords:** ^31^P, qNMR, CRM, Traceability, Certification, Accreditation

## Abstract

Quantitative nuclear magnetic resonance (qNMR) spectroscopy is employed by an increasing number of analytical and industrial laboratories for the assignment of content and quantitative determination of impurities. Within the last few years, it was demonstrated that ^1^H qNMR can be performed with high accuracy leading to measurement uncertainties below 1 % relative. It was even demonstrated that the combination of ^1^H qNMR with metrological weighing can lead to measurement uncertainties below 0.1 % when highly pure substances are used. Although qNMR reference standards are already available as certified reference materials (CRM) providing traceability on the basis of ^1^H qNMR experiments, there is an increasing demand for purity assays on phosphorylated organic compounds and metabolites requiring CRM for quantification by ^31^P qNMR. Unfortunately, the number of available primary phosphorus standards is limited to a few inorganic CRM which only can be used for the analysis of water-soluble analytes but fail when organic solvents must be employed. This paper presents the concept of value assignment by ^31^P qNMR measurements for the development of CRM and describes different approaches to establish traceability to primary Standard Reference Material from the National Institute of Standards and Technology (NIST SRM). Phosphonoacetic acid is analyzed as a water-soluble CRM candidate, whereas triphenyl phosphate is a good candidate for the use as qNMR reference material in organic solvents. These substances contain both nuclei, ^1^H and ^31^P, and the concept is to show that it is possible to indirectly quantify a potential phosphorus standard via its protons using ^1^H qNMR. The same standard with its assigned purity can then be used for the quantification of an analyte via its phosphorus using ^31^P qNMR. For the validation of the concept, triphenyl phosphate and phosphonoacetic acid have been used as ^31^P qNMR standards to determine the purity of the analyte tris(2-chloroethyl) phosphate, and the resulting purity values perfectly overlap within their expanded measurement uncertainties.

## Introduction

Phosphorus plays an important role in physiological processes since it is part of DNA molecules as well as of phosphoric acid esters, e.g., ATP. It is essential in the regulation of metabolism, since phosphorylation and dephosphorylation reactions are rapidly occurring processes at the protein level. Studies on physiological pathways, kinetics, metabolomics, and diseases, but also biomarker discovery, are important fields of investigation [1]. For the analysis of phosphorylation-dephosphorylation processes, several analytical techniques are available, either for qualitative or quantitative detection. These methods include immune assay with antibodies, fluorescence, electrophoresis, label-free detection such as flow cytometry, or stable isotope labeling and mass spectrometry, but also the traditional ^32^P radioactive detection. Prefractionation and enrichment of subfractions still plays an important role in most techniques [2]. Despite all the advantages and disadvantages of the various methods, alternative techniques are always of interest, in particular with regard to quantification. There are only few methods that can use a universal reference standard for quantification, and quantitative nuclear magnetic resonance (qNMR) offers several advantages in this field. The quantification by NMR is based on a signal comparison of the analyte with an internal or external reference standard. In contrast to other methods, e.g., chromatography, the reference standard is independent of the analyte’s chemical structure. Moreover, using primary reference materials, qNMR is a highly accurate method with low measurement uncertainty, also providing traceability to SI units (Système International d’Unités) and thus offering the possibility of certifying reference materials for ^1^H or other nuclei, as for example ^31^P. Over recent decades, the importance of qNMR has significantly increased [3–6]. In 1998 and 2005, Holzgrabe et al. published reviews which covered different general applications of NMR spectroscopy in pharmacy [7] and in particular the application of qNMR [8]. Furthermore, several international pharmacopoeias describe qNMR methods for the determination of the impurity profile of drugs [9]. Jancke et al. delineated NMR spectroscopy as a relative primary analytical method [10], because it can be described completely by mathematical equations, from which a full uncertainty budget may be derived, allowing its employment at the highest metrological level. He also stated that ^1^H NMR spectroscopy is appropriate for quantitative analysis because of the high sensitivity of the proton nuclei combined with relative short relaxation times and virtually 100 % natural abundance. The intensity of the NMR signal is directly proportional to the number of protons that give rise to the signal. Thus, quantification is achieved by measuring the sample proton peak area of interest with respect to a proton signal from an appropriate reference standard, such as an internationally accepted primary certified reference material (CRM) [11]. Using such a primary CRM, e.g., a Standard Reference Material™ (SRM) from the US National Institute of Standards and Technology (NIST), leads to traceability to the SI. There is no need for a reference standard of the same chemical structure as the sample, as it is the case in chromatography or other analytical methods. Several authors described the use of the ^31^P nucleus for quantification by NMR and therefore used different phosphorus-containing references for their experiments, depending on the type of application and solubility, e.g., triphenyl phosphate and sodium phosphate as internal standards [12, 13], or phosphoric acid as external standard in order to avoid reaction with the analyte [14]. Al Deen et al. already recommended the use of CRM for proper traceability and uncertainty budget [13]. This question of traceability in ^31^P qNMR experiments is explicitly addressed within this article. Laboratories working under ISO/IEC 17025 accreditation have to fulfill the demand for traceability to an SI unit. Weber et al. already showed the equivalence of different traceability chains to the NIST SRM benzoic acid and potassium hydrogen phthalate for the ^1^H qNMR method [15]. Since NIST suspended the certification of SRM 1071b (triphenyl phosphate), no phosphorus-containing organic primary CRM from a National Metrological Institute (NMI) is currently available. In contrast, inorganic primary phosphorus CRM exists for water-soluble experiments.

In this paper, we describe the selection and characterization of two candidate organic phosphorus CRM containing both nuclei, ^1^H as well as ^31^P, and their quantification by NMR. The general concept is the quantification of a potential phosphorus standard via its protons using ^1^H qNMR, which is subsequently employed for the quantification of an analyte via its phosphorus using ^31^P qNMR.

## Materials and methods

### Materials

The following standards were purchased from Sigma-Aldrich (% values representing mass fraction purities): triphenyl phosphate (Fluka no.: 05498), phosphonoacetic acid (Fluka no.: 96708), tris(2-chloroethyl) phosphate (Fluka no.: 96382), and dimethyl terephthalate (Fluka no.: 07038, lot BCBL1702V, 99.99 % ± 0.16 %, *k* = 2). The following primary CRM from NIST were used: SRM 350b (benzoic acid, 99.9978 % ± 0.0044 %, *k* = 1.96), SRM 84L (potassium hydrogen phthalate, 99.9934 % ± 0.0076 %, *k* = 2.04), and SRM 194a (ammonium dihydrogen phosphate, 26.93 % ± 0.14 % phosphorus, *k* = 2). Deuterated solvents were also supplied by Sigma-Aldrich: deuterium oxide, D_2_O (Aldrich no.: 151882, 99.9 atom % D); sodium deuteroxide, NaOD (Aldrich no: 372072, 40 % g/g in D_2_O, 99.5 at.% D); dimethyl sulfoxide-*d*_6_, DMSO-*d*_6_ (Aldrich no.: 151874, 99.9 at.% D); chloroform-*d*, CDCl_3_ (Aldrich no.: 151823, 99.8 at.% D); dichloromethane-*d*_2_, CD_2_Cl_2_ (Aldrich no.: 177865, 99.5 at.% D); methanol-*d*_4_, CD_3_OD (Aldrich no.: 151947, 99.8 at.% D); and acetonitrile-*d*_3_, CD_3_CN (Aldrich no.: 151807, 99.8 at.% D).

### Metrological weighing and sample preparation

The weighing processes were performed on an ultra microbalance (UMT 5, Mettler-Toledo GmbH, Switzerland) with a readability of 0.0001 mg, certified by DAkkS (Deutsche Akkreditierungsstelle GmbH) and checked with the International Organization of Legal Metrology (OIML, Paris) class E2 weights. The balance is positioned on a 700 kg stone table, with a U-electrode in place to remove potential static charge. Air buoyancy correction has been taken into account for the mass determination. The ratio of the masses was calculated according to the number of protons in order to ensure approximately 1:1 ratios for the integrals of the calibrant and sample. In most cases, between 10 and 50 mg of substance was weighed out.

For all experiments, seven to ten different samples were prepared by accurately weighing the internal standard and analyte together into an HPLC vial. After adding a suitable deuterated solvent, the samples were thoroughly sonicated to completely dissolve both components; then, the solution was transferred to a 5-mm NMR tube (Schott^®^ NMR sample tubes, professional).

### Preliminary tests

A series of preliminary tests were carried out prior to any quantification experiments by NMR. First, the chemical compatibility between sample and internal standard has been checked by acquiring a proton spectrum and, where required, a ^31^P NMR spectrum of the mixture right after preparation and again after 24 h. To ensure that no impurity lies underneath the peaks of interest, 2D NMR experiments were applied where impurities of less than 0.05 % signal intensity portion can be detected. *T*_1_ relaxation times were evaluated as described in “NMR experiments” since the relaxation time may vary depending on the mixture and the chosen solvent. In a next step, hygroscopy and volatility of the candidate substances were checked since both attributes have a strong influence on the weighing results and thus the outcome of the quantification measurement. A sample was defined to be non-volatile and non-hygroscopic when no change in weighing value of greater than 0.02 mg was obtained over a time period of 10 min. Since the weighing of a single substance takes approximately 1 min, the maximum possible bias for a 10 mg sample amount can be ignored.

### NMR experiments

The experiments for both ^1^H and ^31^P NMR were carried out on a Bruker Avance III 600 MHz spectrometer operating at 600.2 MHz for the proton nucleus and 242.98 MHz for ^31^P. The instrument is equipped with a 5-mm broadband z-gradient probehead. All experiments were performed at 298.2 K while the temperature stability was controlled by a BVT 3200 unit.

For qualitative ^1^H NMR measurements, a standard single pulse experiment with 16 scans, a flip angle of 30°, and a relaxation delay (*D*_1_) of 2 s were utilized and the spectral width was set to 22 ppm with 65,536 data points, and for ^31^P NMR, a single-pulse sequence was used with power-gated decoupling and a flip angle of 30°. Sixty-four scans were acquired with a relaxation delay of 4 s, a spectral width of 395 ppm, and 65,536 data points.

Determination of the *T*_1_ relaxation time for protons was carried out using the inversion recovery experiment which was performed in automation mode using 11 different delays that ranged from 0.01 to 20 s, and the relaxation delay was set mostly to 30 s and the number of scans four or higher when needed. In total, 65,536 data points were acquired. *T*_1_ relaxation time for ^31^P nuclei was also determined by inversion recovery experiments. Nine different delays ranging from 0.01 to 5 s were utilized; the relaxation delay was set to 20 s and the number of scans to eight or higher when required. For evaluation of the *T*_1_ relaxation times, the *T*_1_/*T*_2_ relaxation module of the software TopSpin 2.1 was used with non-linear fit of the peak intensities. Inversion recovery experiments were applied for each single compound along with the mixture of analyte and internal standard.

Proton quantitative spectra were acquired with seven to ten different samples for each qNMR series. A number of 16 transients with 65,536 data points each were collected to ensure a signal to noise ratio of >300 for the relevant peaks, with a standard single pulse experiment without decoupling. To receive fully relaxed NMR spectra with maximum signal intensity, a 90° pulse was applied. Based on previous *T*_1_ inversion recovery experiments, the *T*_1_ relaxation delay was checked for each mixture and set accordingly. For all ^1^H experiments, a relaxation delay of 60 s was chosen, representing a minimum of seven times the longest *T*_1_ in the mixture.

For ^31^P qNMR measurements, inverse gate proton decoupling during data acquisition was applied to minimize signal enhancement due to the nuclear Overhauser effect with a flip angle of 30°. A relaxation delay of 10 s was set for all ^31^P qNMR experiments which also guarantees at least seven times the longest *T*_1_ relaxation time. Each spectrum was acquired with 64 scans and 65,536 data points.

The transmitter frequency offset (*O*_1_) was always set in such a way that neither calibrant signal nor sample signal was affected in order to avoid saturation. All experiments were carried out under non-spinning conditions with regard to the high magnetic field to avoid spinning side bands. Prior to Fourier transformation, a window function was applied and the spectra were processed with a line broadening of 0.1 Hz for proton spectra and 3 Hz for ^31^P spectra, respectively, and zero filling was done once. After careful manual phasing and baseline correction, the integration of the signals was carried out manually. The integration of calibrant signal and sample signal was always done in the same way in regard to the line width of calibrant and sample signal with both signals integrated with or without ^13^C satellites. For ^31^P qNMR, single-bond ^13^C satellites were only present in the case of phosphonoacetic acid and became part of the integral. Referring to this, other ^31^P signals without ^13^C satellites were integrated based on multiples of their half width.

## Results and discussion

### Selection of candidate substances

**Fig. 1:**
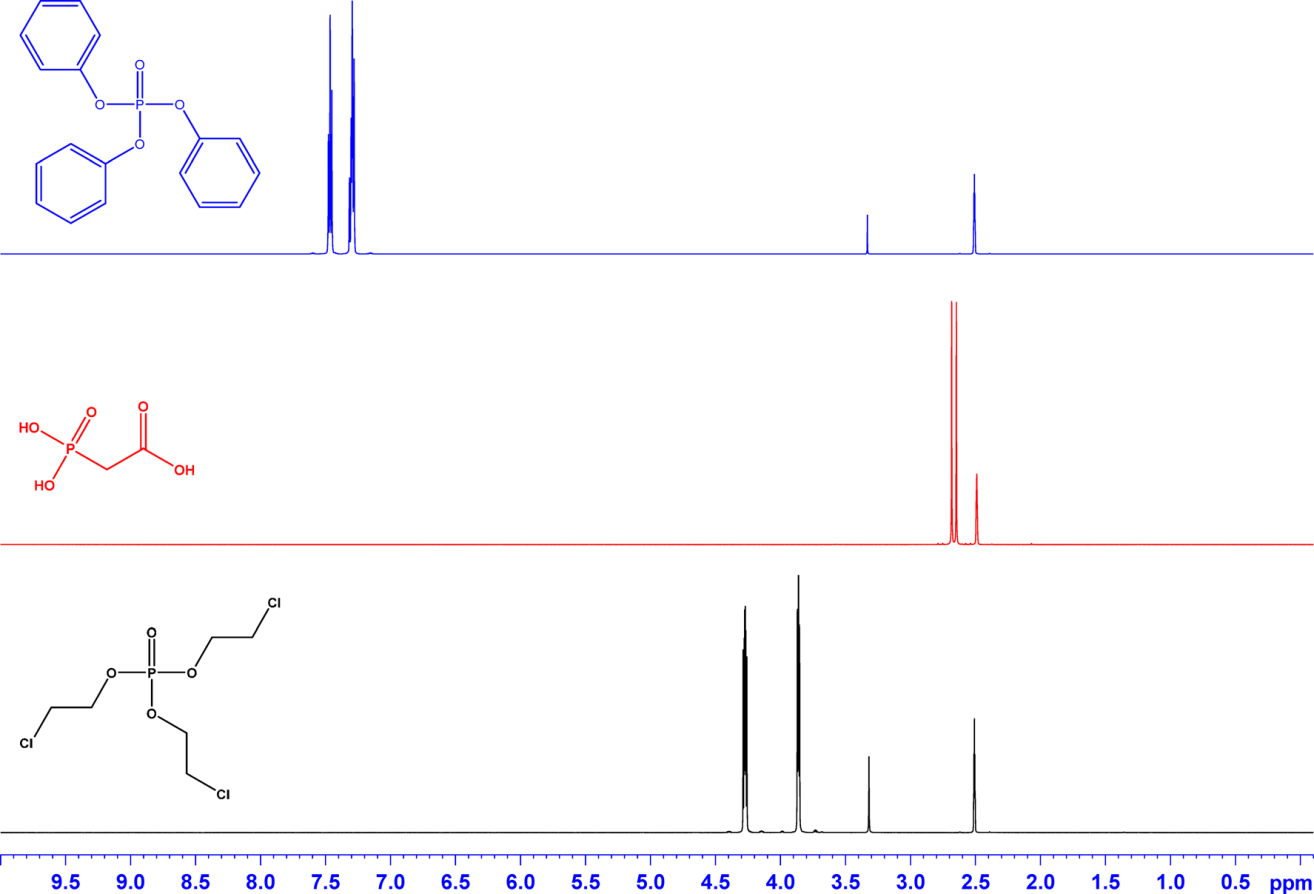
^1^H NMR spectra for triphenyl phosphate (*blue*), phosphonoacetic acid (*red*), and tris(2-chloroethyl) phosphate (*black*). All substances were dissolved in DMSO-*d*_6_

**Fig. 2:**
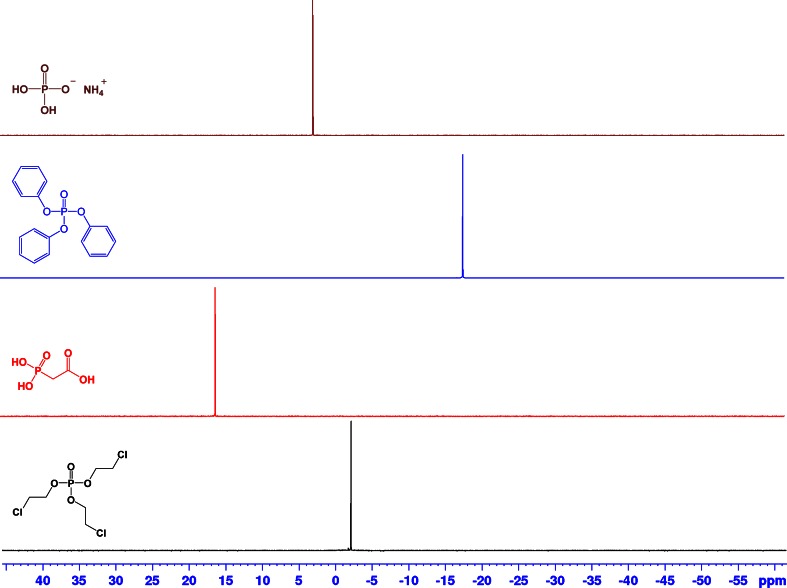
^31^P NMR spectra for ammonium dihydrogen phosphate in D_2_O (*brown*), triphenyl phosphate in CDCl_3_ (*blue*), phosphonoacetic acid in D_2_O (*red*), and tris(2-chloroethyl) phosphate in CDCl_3_ (*black*)

Based on various parameters, including solubility, structure, chemical shifts, stability, homogeneity, and purity, two different organic compounds have been selected as candidates for their use as ^31^P qNMR references, triphenyl phosphate and phosphonoacetic acid. As a prerequisite to the concept of certification, they carry both types of nuclei, ^1^H and ^31^P. Triphenyl phosphate was chosen as a standard that can be applied for measurements in organic solvents, whereas phosphonoacetic acid can be used in aqueous solutions. Tris(2-chloroethyl) phosphate was selected as phosphorus-containing analyte in order to show the proof of concept in a ^31^P qNMR application. In order to overcome restrictions in solubility, the molecules have further been tested for solubility in other solvents that are typically used in NMR experiments (see Fig. 4). For their subsequent use in qNMR analyses, one of the major requirements is that the signals of the internal reference standard and the analyte do not overlap within the mixture. This applies for those ^1^H signals and the ^31^P signals respectively used for quantification. For visualization of the respective peak positions, the corresponding ^1^H and ^31^P NMR spectra of the two candidates and the analyte have been measured separately (Figs. 1 and 2).

Figure 1 shows the qualitative characterization of the three substances using ^1^H NMR spectroscopy. Triphenyl phosphate displays two signals in the aromatic region at 7.5 ppm with one set of signals at 2.5 and 3.3 ppm coming from the solvent DMSO-*d*_6_ and its residual water, respectively. Phosphonoacetic acid has signals only in the high-field region at 2.7 ppm, the other peak in the spectrum is deriving from the solvent DMSO-*d*_6_, and the signal generated by the water in DMSO-*d*_6_ is shifted to low field (∼12 ppm) due to the hydrogen bonds with the acetic group of the phosphonoacetic acid. Tris(2-chloroethyl) phosphate shows signals at 3.8 and 4.3 ppm, again with DMSO-*d*_6_ and water at 2.5 and 3.3 ppm. It is important to note that the signals of triphenyl phosphate and phosphonoacetic acid are well separated from the tris(2-chloroethyl) phosphate signals, thus allowing the quantification of the analyte tris(2-chloroethyl) phosphate via ^1^H qNMR. Despite the fact that the spectra of the single substances shown in Fig. 1 were measured in DMSO-*d*_6_, the choice of another suitable solvent for a subsequent qNMR measurement is perfectly possible, according to the data given in Fig. 4.

Figure 2 shows the ^31^P NMR spectra of ammonium dihydrogen phosphate (NIST SRM 194a), the two candidate phosphorus standards triphenyl phosphate and phosphonoacetic acid, and the analyte tris(2-chloroethyl) phosphate. Ammonium dihydrogen phosphate is one of the few primary inorganic phosphorus standards that can be used in ^31^P qNMR, with the limitation that it is only water soluble. Each spectrum shows only one single peak, ammonium dihydrogen phosphate at 3 ppm in D_2_O, triphenyl phosphate at −18 ppm in chloroform-*d*, phosphonoacetic acid at 15 ppm in deuterium oxide, and tris(2-chloroethyl) phosphate at −2 ppm in chloroform-*d*. Thus, in the case of ^31^P qNMR, it is possible to combine ammonium dihydrogen phosphate and phosphonoacetic acid in an aqueous mixture with well-separated signals. In addition, the candidate phosphorus CRM triphenyl phosphate and phosphonoacetic acid can both be combined with the analyte tris(2-chloroethyl) phosphate for subsequent measurements. Again, the choice of a suitable solvent for the respective mixture is a prerequisite for the qNMR experiment using an internal standard.

### Measurement concept for ^31^P qNMR CRM

Wherever possible, the traceability of an assigned purity value of ^31^P qNMR CRM should be realized based on phosphorus nuclei since the reference standard is subsequently used in the same way; nevertheless, there can be specific reasons why this requirement is difficult to fulfill.

Water-soluble and phosphorus-containing reference standards are widely available, e.g., NIST SRM 194a (NH_4_H_2_PO_4_) or NIST SRM 200b (KH_2_PO_4_), which can be used as primary standard for establishing traceability in ^31^P qNMR measurements. Since such substances are not readily soluble in common organic solvents, as DMSO or methanol, an organic phosphorus-containing internationally accepted reference standard (e.g., a primary CRM from a NMI) is needed. For many years, NIST provided triphenyl phosphate (SRM 1071b) which could be used for establishing traceability. Unfortunately, this reference material is no longer available and no other NMI offers a comparable CRM. Therefore, direct traceability to a NIST SRM, as in the case of the water-soluble CRM candidate phosphonoacetic acid, is not possible for triphenyl phosphate so quantification has to be realized by ^1^H qNMR measurements.

**Fig. 3:**
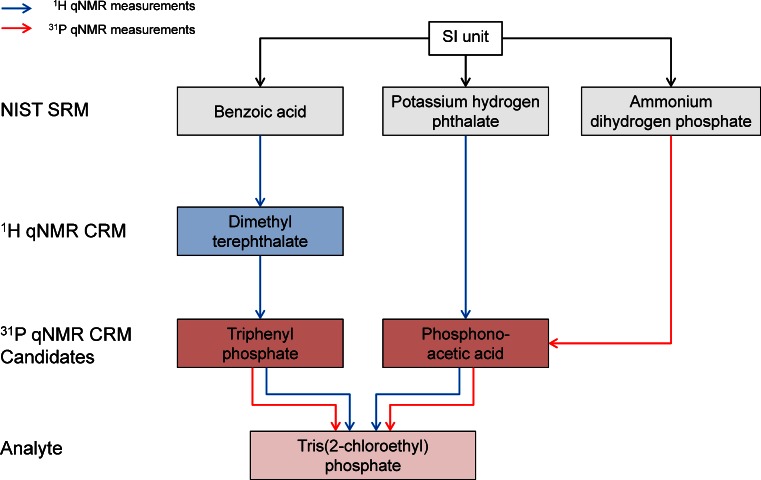
Traceability chains for the purity determination of ^31^P qNMR standards triphenyl phosphate and phosphonoacetic acid. The quantification of phosphonoacetic acid using two independent NIST reference materials and different NMR experiments demonstrates the proof of concept that the received purity value is independent of the nucleus. This concept may be applied for the quantification of triphenyl phosphate. In a subsequent step, purity determination of tris(2-chloroethyl) phosphate is performed by ^31^P qNMR using triphenyl phosphate and phosphonoacetic acid as internal standards. The *blue arrows* symbolize ^1^H qNMR measurement and *red arrows* ^31^P qNMR measurements

Figure 3 shows the concept for the quantification of the two different phosphorus candidate CRM. Using ammonium dihydrogen phosphate (NIST SRM 194a) in a ^31^P qNMR measurement, the obtained purity value of phosphonoacetic acid represents the first purity of a phosphorus-containing product, which is traceable to NIST SRM and hence to SI unit. Phosphonoacetic acid is additionally employed in a ^1^H qNMR measurement with potassium hydrogen phthalate (NIST SRM 84L) as reference as it is crucial to show that ^1^H qNMR results and ^31^P qNMR results are consistent within their measurement uncertainties for the quantification of triphenyl phosphate. Once these individual traceability chains prove the independence from the nuclei, triphenyl phosphate can be quantified according to the traceability chain on the left-hand side of the figure using only ^1^H qNMR measurements.

As a last step, the purity of tris(2-chloroethyl) phosphate is measured in a control experiment in order to confirm the prior postulation and the whole concept, using the purities of triphenyl phosphate (obtained in organic solvent, traceable to benzoic acid) and phosphonoacetic acid (obtained in water, traceable to potassium hydrogen phthalate or ammonium dihydrogen phosphate).

### Experimental prerequisites

**Fig. 4:**
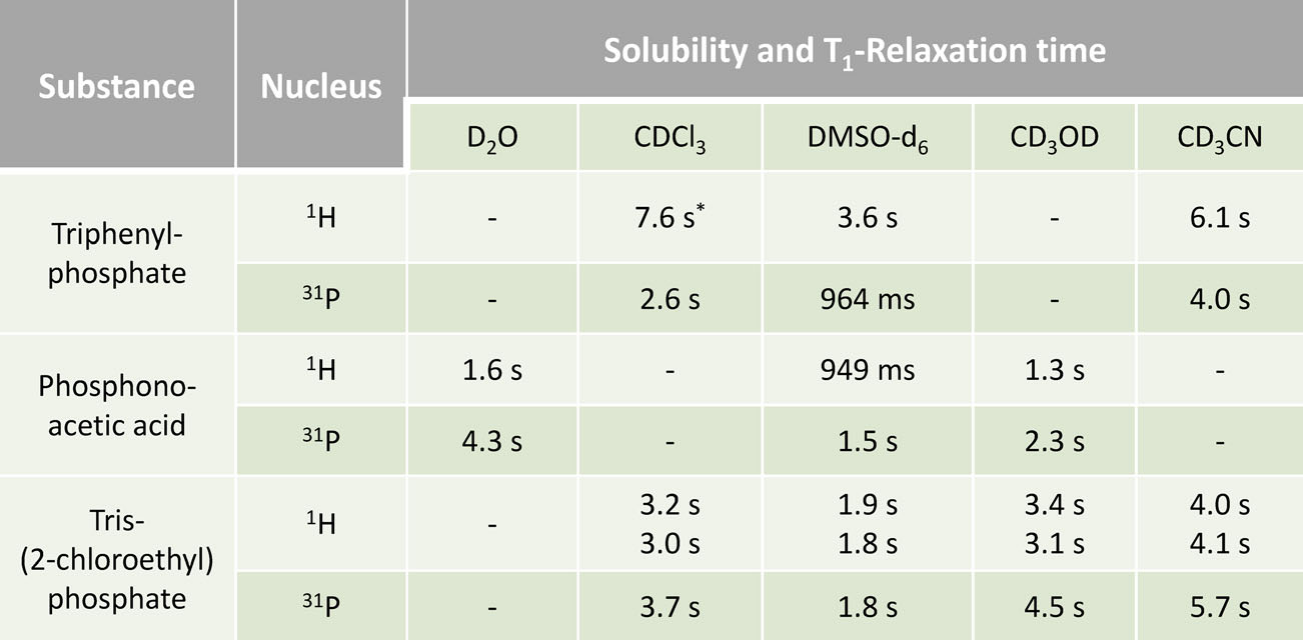
Table of deuterated solvents in which the ^31^P CRM candidates triphenyl phosphate, phosphonoacetic acid, and the analyte tris(2-chloroethyl) phosphate are soluble and corresponding relaxation times (*T*_1_) for both nuclei, ^1^H and ^31^P. In the *boxes* which do not display time specifications, the substance was not sufficiently soluble. *Due to overlaps with the CDCl_3_ signals, the solvent CD_2_Cl_2_ was used as a substitute

In order to demonstrate compatible combinations of analyte and standard, a number of preliminary tests were performed, as described in “Preliminary tests.” It was necessary to exclude potential signal overlaps, as well as reactions between analyte and standard or reactions with the solvent. For the determination of a suitable deuterated solvent, the tolerance between analyte and standard within one sample was measured by running ^1^H NMR experiments at *t* = 0 h (immediately after sample preparation) and *t* = 24 h. The spectra measured from both time points were compared by electronic overlay, and no differences could be observed. Moreover, it was confirmed that the substances under examination had no hydrophilic or volatile character and showed a simple signal pattern in order to allow clear spectra of mixtures without interference by potential impurities. Prior to the quantification measurements, the *T*_1_ relaxation times in different deuterated solvents were determined by inversion recovery experiments, as described in “NMR experiments.” Relaxation times should preferably be short, in view of adjusting the relaxation delay to at least seven times *T*_1_. Figure 4 shows in which deuterated solvents the single candidate substances are soluble and lists the corresponding relaxation times for both nuclei, ^1^H and ^31^P. For some substances, the table shows more than one value for relaxation times due to multiple protons within the structure having different relaxation behavior.

Since the indicated relaxation times may slightly vary in the mixture, the longest value in a mixture is used for calculating the overall relaxation time of the experiment. For the *T*_1_ determination of triphenyl phosphate, CDCl_3_ could not be used due to overlapping of the sample with the solvent signal. For this reason, CD_2_Cl_2_ was used as an alternative. As relaxation times largely depend on the mixture and the solvent, the evaluation of the relaxation times was carried out for entire mixtures (internal standard, analyte, and solvent) simultaneously with the compatibility check.

### Quantification of the candidate CRM phosphonoacetic acid

To prove the concept that no bias in a qNMR measurement is created when the purity is determined either using proton or phosphorus signals, the quantification of phosphonoacetic acid was performed by ^31^P and ^1^H qNMR measurements. In a first experiment, the purity of phosphonoacetic acid was determined by ^31^P qNMR using ammonium dihydrogen phosphate (NIST SRM 194a) as a reference, resulting in a purity value of 99.26 % ± 0.75 %. The second purity determination was carried out by ^1^H qNMR measurement using potassium hydrogen phthalate (NIST SRM 84L) as a reference, resulting in a purity value of 99.32 % ± 0.17 %.

**Fig. 5:**
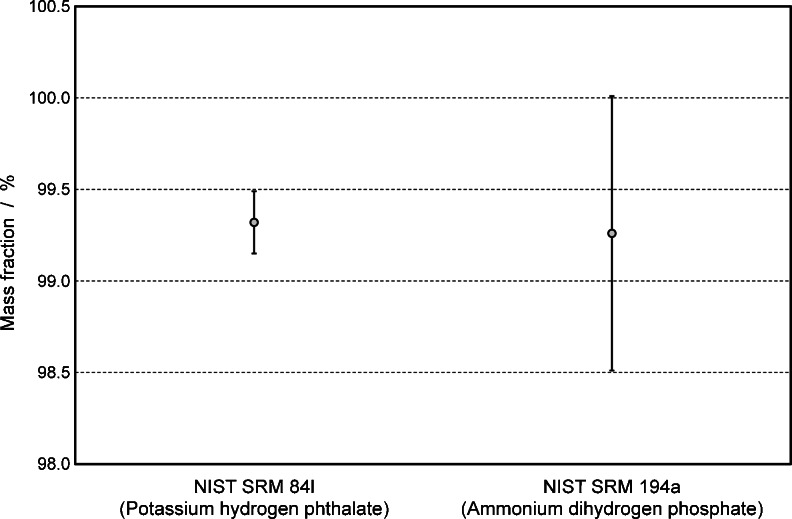
Graphical illustration of purity values and their expanded uncertainties for phosphonoacetic acid. Two different primary references from NIST were used for establishing traceability to SI unit. Potassium hydrogen phthalate was used as internal standard in ^1^H qNMR measurements and ammonium dihydrogen phosphate in ^31^P qNMR measurements

These two results deriving from two independent traceability chains and via qNMR measurements of different nuclei are fully consistent and overlap within their expanded measurement uncertainties, as shown in Fig. 5. The observed measurement uncertainties for the ^31^P qNMR measurements are significantly higher compared to the ^1^H qNMR values due to effects described in “Measurement uncertainty.”

### Quantification of the candidate CRM triphenyl phosphate

After the ^1^H and ^31^P qNMR experiments for the quantification of phosphonoacetic acid proved the independence of the received purity value from the nucleus, triphenyl phosphate could be quantified by ^1^H qNMR. Direct application of benzoic acid as standard reference material in proton NMR was not feasible due to the overlapping of the signals in the aromatic region. Therefore, the traceability chain was achieved by the use of the CRM dimethyl terephthalate as an internal reference standard and which is traceable to NIST SRM 350b, benzoic acid. The purity of triphenyl phosphate was determined to be 99.95 % ± 0.22 %. Although this value was achieved by ^1^H qNMR measurements, it can be employed in subsequent ^1^H and ^31^P qNMR measurements as proven above. To remove any doubt regarding this conclusion, the concept was proven in a further step, in which a proton and phosphorus-containing sample was chosen as sample for the quantification by the two ^31^P qNMR CRM candidates.

### Quantification of tris(2-chloroethyl) phosphate by ^31^P qNMR

In the previously described certification concept for phosphorus standards, the chosen candidate compounds contain both nuclei, ^1^H and ^31^P. After triphenyl phosphate and phosphonoacetic acid have been quantified via their protons and/or phosphorus, the resulting purity values were used for the subsequent purity determination by ^31^P qNMR experiments. The quantification of tris(2-chloroethyl) phosphate on the basis of ^31^P qNMR yielded purities of 98.43 % ± 0.66 % by using triphenyl phosphate as a reference and 98.45 % ± 0.44 % by using phosphonoacetic acid as a reference. Additionally, the quantification of tris(2-chloroethyl) phosphate on the basis of ^1^H qNMR yielded purities of 98.41 % ± 0.53 % by using triphenyl phosphate as a reference and 98.88 % ± 0.52 % by using phosphonoacetic acid as a reference.

**Fig. 6:**
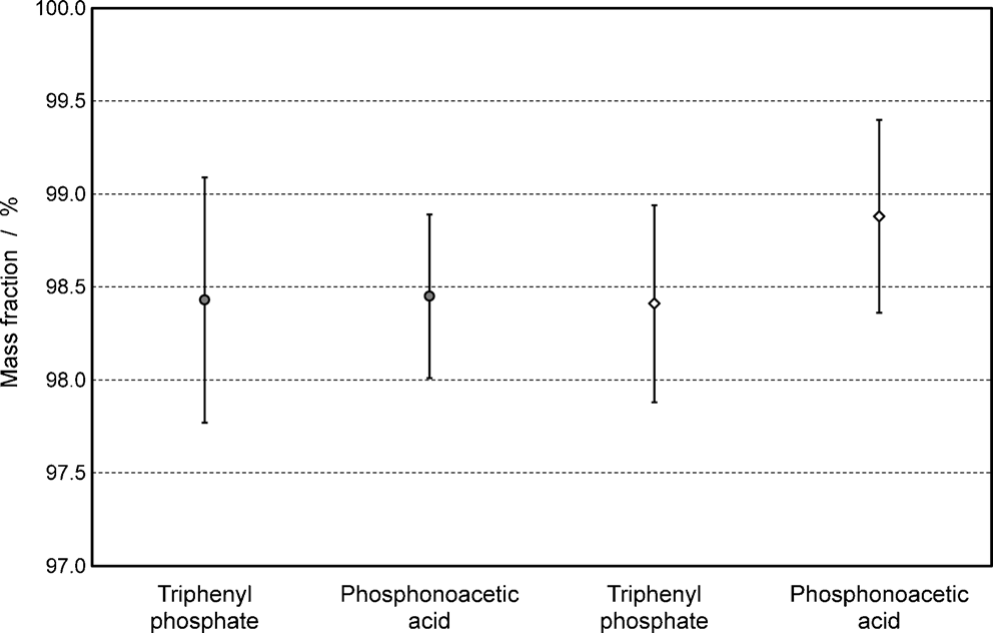
Graphical illustration of purity values and expanded measurement uncertainties for tris(2-chloroethyl) phosphate. Phosphonoacetic acid and triphenyl phosphate are used as internal standards, and the experiments are based on ^31^P (*circle*) and ^1^H (*diamond*) qNMR measurements

All these results derived from different nuclei and different traceability chains are summarized in Fig. 6, and the consistency of the data is demonstrated by the overlap of their expanded measurement uncertainties.

A potential combination of triphenyl phosphate and phosphonoacetic acid for quantification within the same NMR tube was not possible due to their different solubility (polar and non-polar). It is important to note that the experiments are independent of each other, and different measurement systems had to be tested in each case to find a suitable reference standard, selecting the appropriate deuterated solvent and elaborating the appropriate acquisition parameters, e.g., relaxation times (see Fig. 4).

### Measurement uncertainty

The quantifications of the purity in the shown experiments are based on the so-called internal standard method where the analyte signal is directly compared with an internal reference signal. This approach can not only be applied for ^1^H but also for ^31^P signals. The following equation shows the relevant parameters for the calculation of the CRM purity, which is finally expressed as percent mass fraction.

1 $$ {P}_{\mathrm{S}}=\frac{I_{\mathrm{A}}}{I_{\mathrm{Ref}}}\cdot \frac{N_{\mathrm{Ref}}}{N_{\mathrm{A}}}\cdot \frac{M_{\mathrm{A}}}{M_{\mathrm{Ref}}}\cdot \frac{m_{\mathrm{Ref}}}{m_{\mathrm{S}}}\cdot {p}_{{}_{\mathrm{Ref}}} $$

*I*_A_: Integral area of the analyte signal
*I*_Ref_: Integral area of the reference signal
*m*_S_: Mass of the sample (g)
*m*_Ref_: Mass of the reference (g)
*M*_A_: Molecular mass of the analyte (g/mol)
*M*_Ref_: Molecular mass of the reference (g/mol)
*N*_A_: Number of nuclei generating the analyte signal
*N*_Ref_: Number of nuclei generating the reference signal
*P*_S_: Purity of the sample as mass fraction (g/g)
*P*_Ref_: Purity of the reference as mass fraction (g/g)

It should be noted that even minor uncertainty contributions from air buoyancy correction were taken into account. Therefore, climate data were recorded during each weighing step.

The uncertainty calculation is based on well-established guidelines [16, 17]. For the purity determination, the combined standard uncertainty *u*_c_(*P*_s_) can be calculated by Eq. (2):

2 $$ {u}_{\mathrm{c}}\left({P}_{\mathrm{S}}\right)={P}_{\mathrm{S}}\sqrt{u_{\mathrm{rel}}^2\left({m}_{\mathrm{Ref}}\right)+\kern0.5em {u}_{\mathrm{rel}}^2\left({m}_{\mathrm{S}}\right)+{u}_{\mathrm{rel}}^2\left({P}_{\mathrm{Ref}}\right)+{u}_{\mathrm{rel}}^2\left({M}_{\mathrm{A}}\right)+{u}_{\mathrm{rel}}^2\left({M}_{\mathrm{Ref}}\right)+{u}_{\mathrm{rel}}^2\left({I}_{\mathrm{Ind}}\right)+{u}_{\mathrm{rel}}^2\left(\mathrm{Rep}\right)} $$

**Fig. 7:**
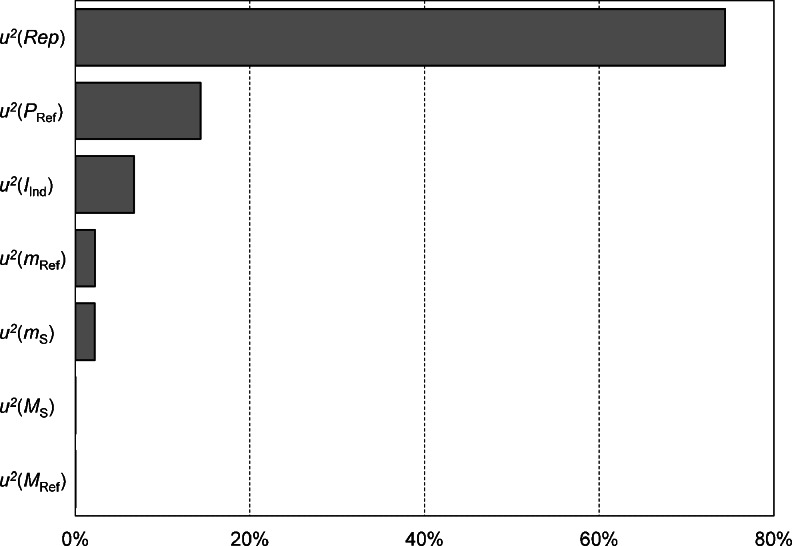
Overview on typical contributions to the relative standard uncertainty (relative squared contributions are given) for the quantification of tris(2-chloroethyl) phosphate using phosphonoacetic acid as internal standard in a ^31^P qNMR measurement

The combined standard uncertainty is determined by statistical as well as systematic contributions whereby the statistical contribution *u*(Rep) arises from the repeatability of weighing and signal integration. On the other hand, various systematic contributions, e.g., the air buoyancy correction, balance parameters, molecular masses, and the purity of the reference (expressed as a mass fraction), were taken into account. Uncertainty contributions were increased if the sample weight was smaller than the recommended minimum sample weight. Mass determination uncertainty (weighing and air buoyancy correction) was calculated according to published literature [18], and no further details are given in this article. Phase correction and the integration of the signals were done manually and may slightly differ with the operator. This individual influence is considered in the overall uncertainty budget as “individual integration contribution” *u*(*I*_Ind_). This uncertainty *I*_Ind_ was carefully evaluated and was calculated based on a series of experiments where different operators analyzed various sets of analysis data at different points in time. As shown in Fig. 7, the overall repeatability of the measurement represents the most significant uncertainty contribution in ^31^P qNMR measurements.

^31^P has a relative receptivity of 0.0665 that means it is less sensitive than ^1^H (receptivity of 1.00) thereby leading to a lower signal to noise ratio, which can partially be compensated by a higher analyte concentration or a higher number of scans, but it has to be considered that higher analyte concentrations can lead to reduced solubility and therefore higher viscosity or broadening of the signals.

## Conclusions

In the described new certification concept for ^31^P qNMR CRM, it has been successfully shown how traceability to SI unit can be established by qNMR using different nuclei. Within this concept, it was proven that purity values of a single material (phosphonoacetic acid) using ^1^H or ^31^P measurements are fully consistent with each other. Taking advantage of this concept, the purity of triphenyl phosphate was determined by ^1^H qNMR, but the product with its purity value was subsequently used as ^31^P qNMR CRM.

In an additional experiment, the purity of an exemplary analyte (tris(2-chloroethyl) phosphate) was measured following different traceability chains and solvent systems. Since the results are comparable within the range of their measurement uncertainties, the robustness of the certification concept is demonstrated. A possible application for the usage of these ^31^P qNMR CRM is given by the measurements of tris(2-chloroethyl) phosphate.

The work described in this article represents an important step towards a successful method validation for ^31^P qNMR measurements. The requirements for a reference material producer under ISO Guide 34 accreditation cover additional data such as homogeneity of the material and short-term and long-term stability [19]. The two described ^31^P qNMR CRM candidates triphenyl phosphate and phosphonoacetic acid are undergoing these additional procedures, and it is expected that new CRM will soon be available to the analytical community.
